# Supplementary figures and images for: Natural variation in infection specificity of Caenorhabditis briggsae isolates by two RNA viruses
Source: PLoS Pathog. 2024 Jun 11;20(6):e1012259. doi: 10.1371/journal.ppat.1012259 (PMC11195985; doi:10.1371/journal.ppat.1012259)

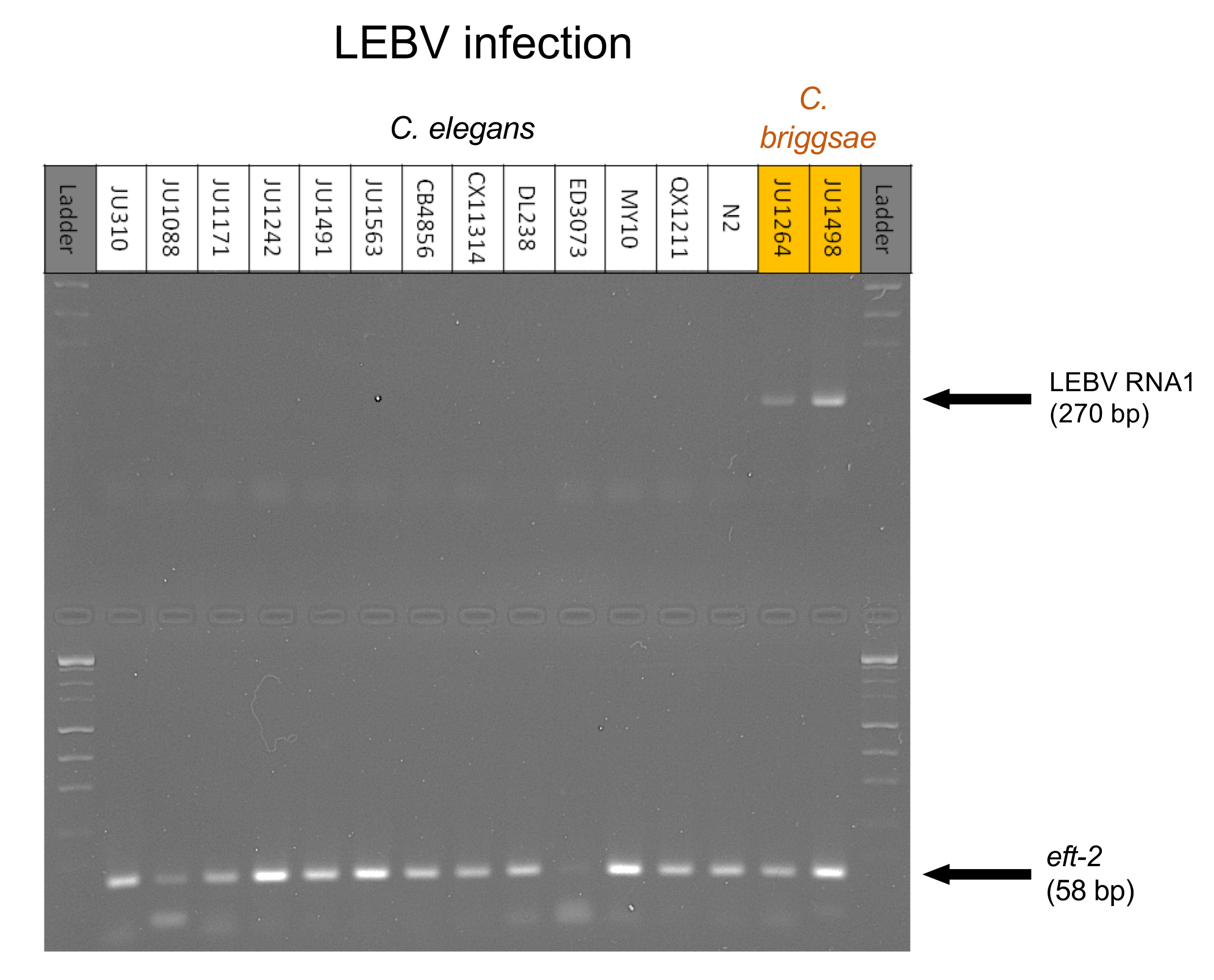

Supplement: S1 Fig — Electrophoresis on an agarose gel showing the results of a RT-PCR for LEBV RNA1. The inoculated strains are indicated on top of the gel. The infection was performed as in Fig 2A. The C. elegans strains include some that are infected at high levels by the Orsay virus, such as JU1491, JU1563, DL238, JU1242. A positive control for the LEBV inoculate is shown with the two C. briggsae strains JU1264 and JU1498. A positive RT-PCR control with eft-2 is shown on the bottom gel. (TIF) [file ppat.1012259.s001.tif]

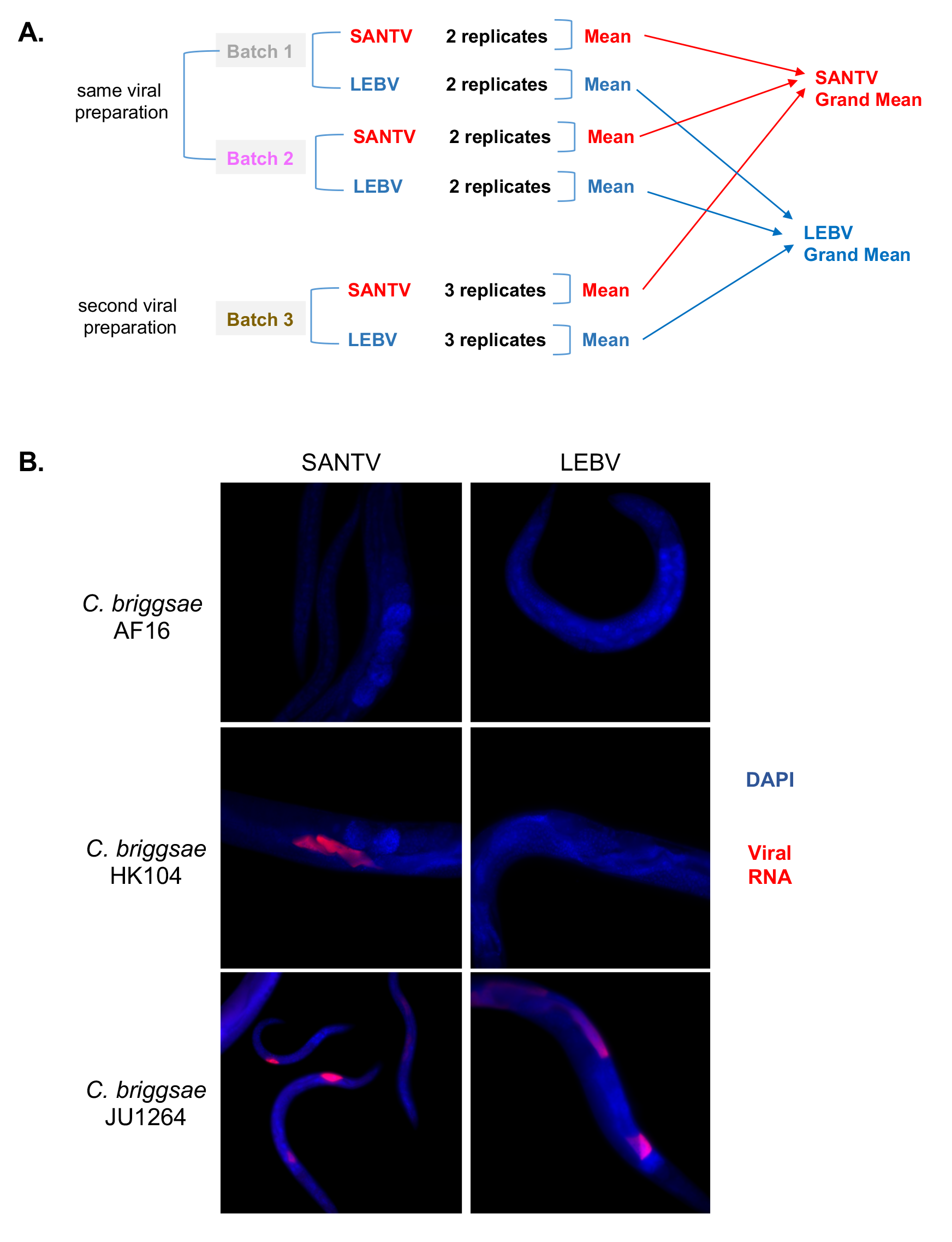

Supplement: S2 Fig — (A) Design for the assays of the C. briggsae wild isolates. The data for each replicate (cf. Methods) are shown in S3 Table and the grand mean in Fig 2. The absolute level of infection differs among batches but the results are generally consistent. (B) Representative FISH images of the viruses, here using RNA1 probes for each virus. The images were acquired in the DAPI and FISH channels using a 40x objective and super-imposed in false colors. (TIF) [file ppat.1012259.s002.tif]

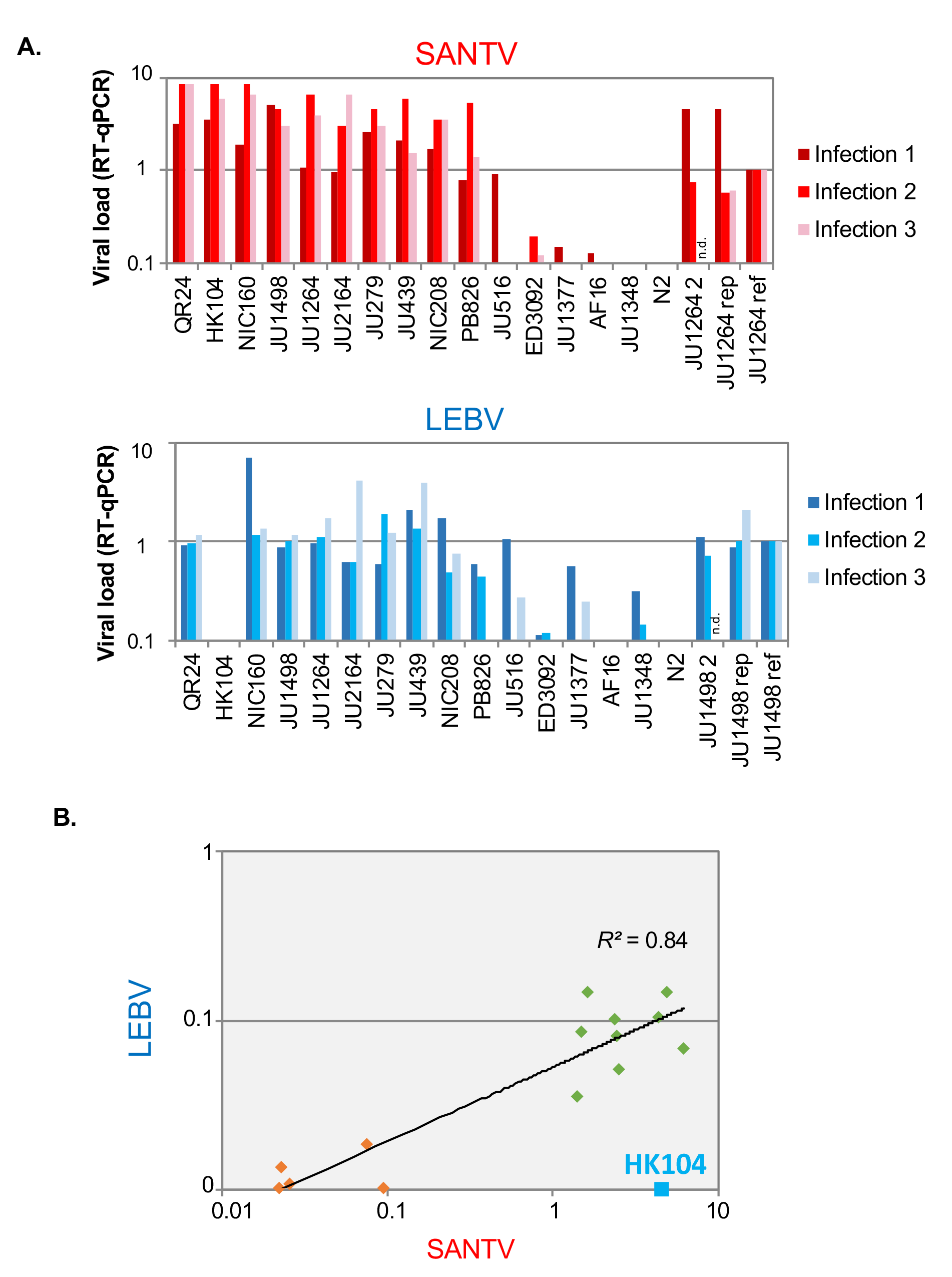

Supplement: S3 Fig — (A) Viral infections of a subset of C. briggsae isolates, with the viral load assayed by RT-qPCR for RNA1 of the corresponding virus. C. elegans N2 is a negative control for the initial viral inoculum. The plotted viral load corresponds to the ratio between the amplification of viral RNA1 over that of eft-2, normalized by that in the reference infection (JU1264 for SANTV, JU1498 for LEBV). The three infection replicates were performed in parallel. C. elegans N2 was used as a negative control. The control strains for each virus were tested twice per infection (JU1264 and JU1264-2 for SANTV; JU1498 and JU1498-2 for LEBV). In the first infection, two RNA preparations of the control strain were conserved and reused in the RT-qPCR for comparison among experiments. One was designated as reference (JU1264-ref and JU1498-ref) and the other served to assess the repeatbility of the RT-qPCR (JU1264-rep and JU1498-rep). (B) Two-dimensional plot displaying the mean between the three replicates. HK104 is an outlier. The other strains show a good correlation between their sensitivity to SANTV and LEBV (regression line and correlation excluding here HK104). A larger set of C. briggsae isolates was assayed by FISH in Fig 2. (TIF) [file ppat.1012259.s003.tif]

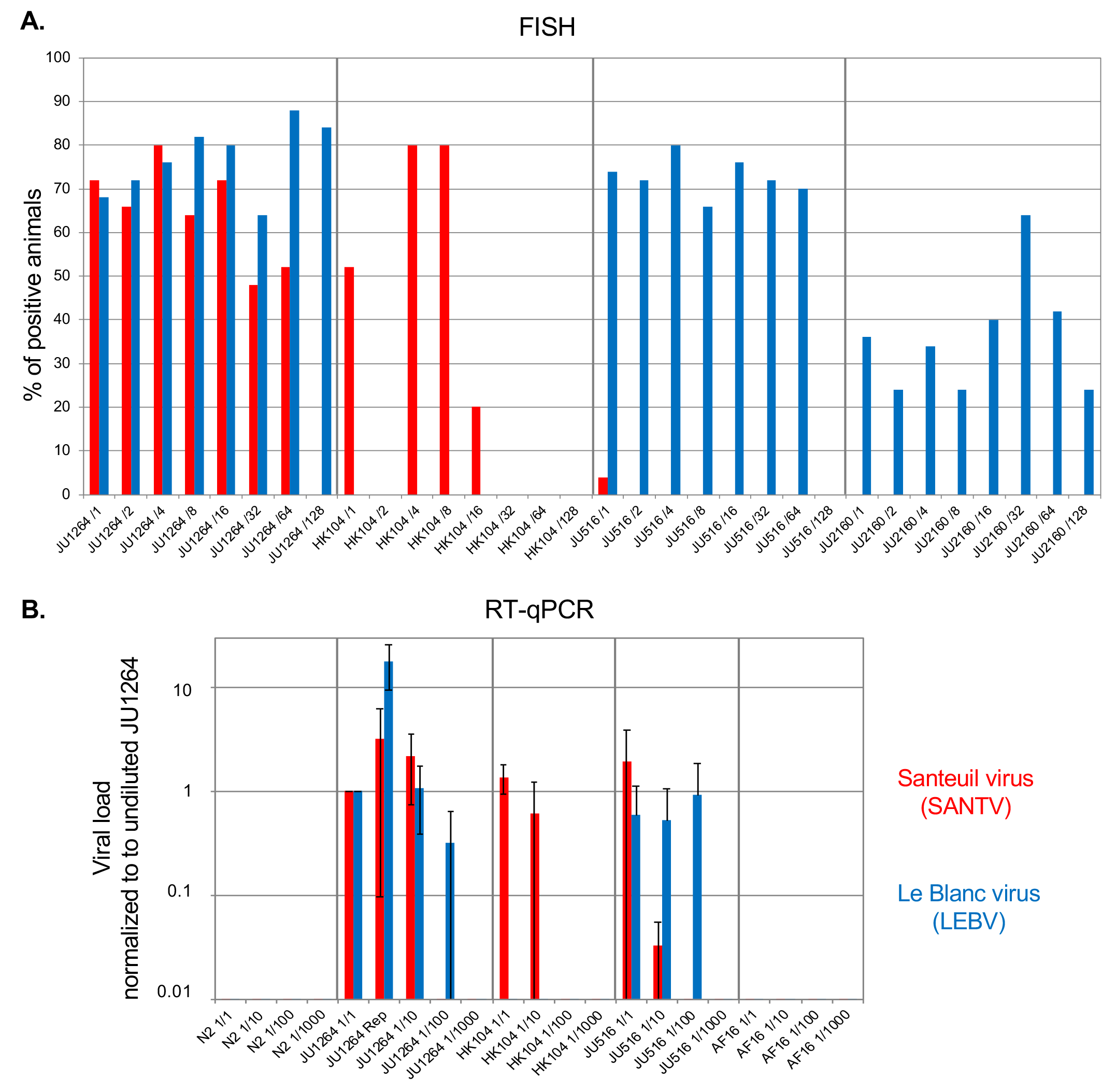

Supplement: S4 Fig — In both panels, SANTV infections are shown in red, LEBV infections in blue. (A) Proportion of infected animals as assayed by FISH staining. Four C. briggsae isolates were infected with successive two-fold dilutions of the same viral preparations of either SANTV JUv1264 or LEBV JUv1498, using RNA1 probes for each virus. 50 animals were scored per condition. The infection was initiated by inoculating a plate containing 5 L4 larvae and transferring 20 L4 larvae of the F1 generation, and assaying adults 3 days later, at 23°C. (B) Viral load assayed using RT-qPCR, in a separate infection experiment. C. elegans N2 is used as a control for amplification of the viral inoculum. The undiluted viral preparations on JU1264 are used to normalize and are indicated as "JU1264 1/1". A separate replicate was performed and indicated as "JU1264 Rep". In each condition, n = 2 RT-qPCR replicates for SANTV, 3 replicates for LEBV. Bars show standard deviation. (TIF) [file ppat.1012259.s004.tif]

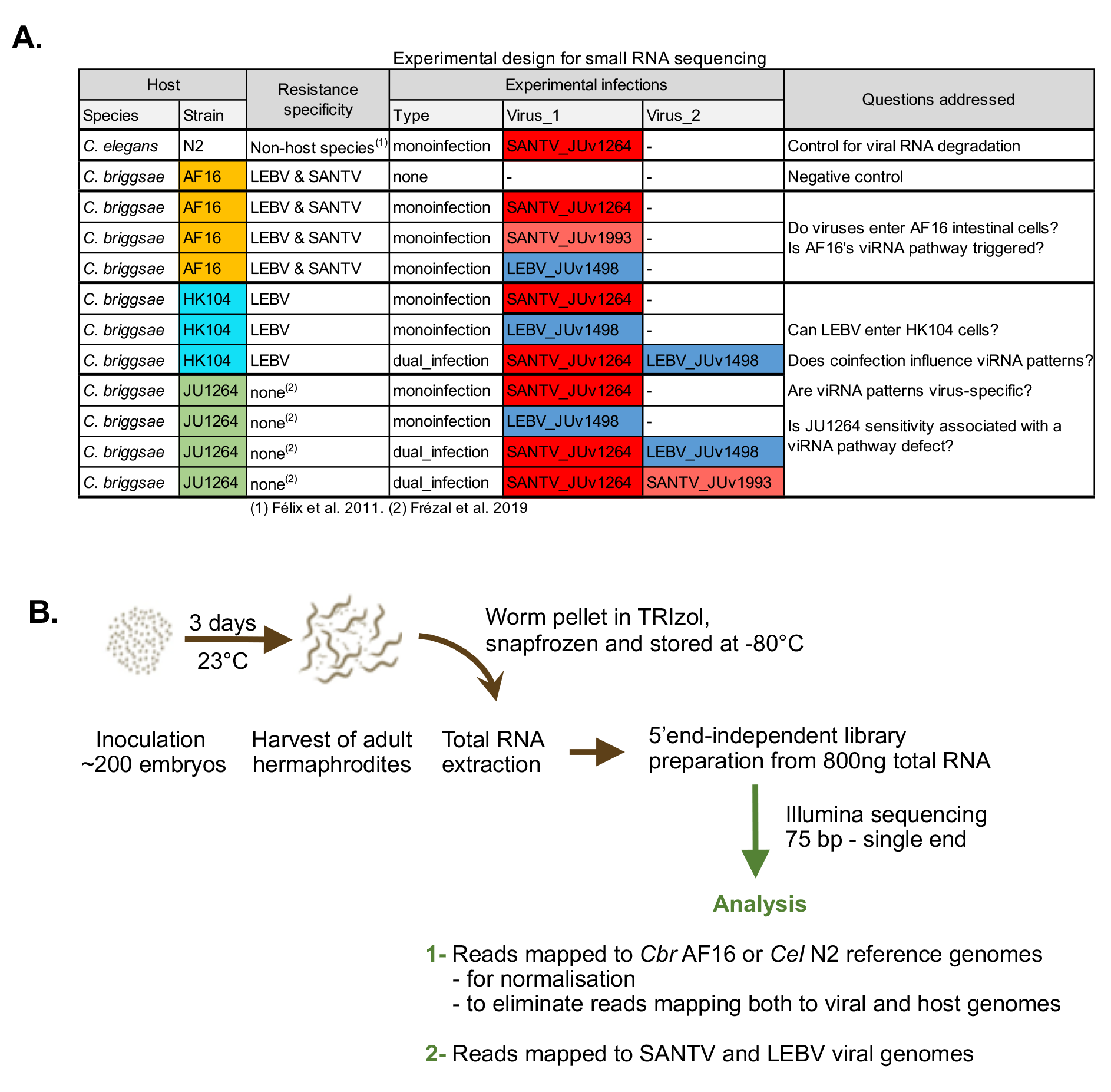

Supplement: S5 Fig — (A) Details of experimental conditions and questions addressed. Embryos were obtained by bleaching gravid hermaphrodites. The strain names are color coded as in Fig 3; the virus names are color coded as in Fig 1. The SANTV variant JUv1993 was also used; this variant tends to outcompete JUv1264 in long co-infection experiments (Frézal et al. 2019 [16]). (B) Schematic overview of the experimental flow. (TIF) [file ppat.1012259.s005.tif]

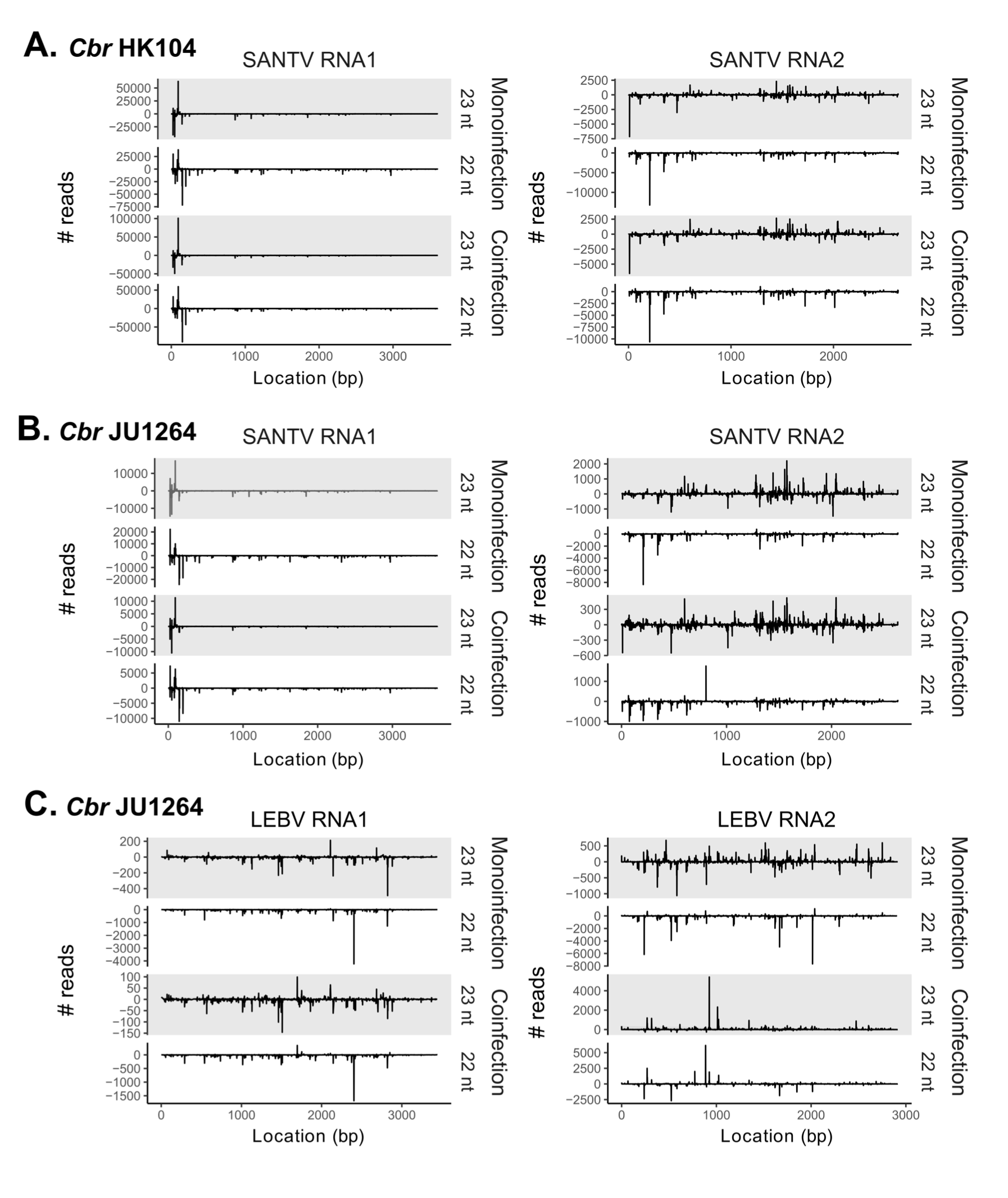

Supplement: S6 Fig — (A) C. briggsae HK104 infected with SANTV JUv1264. (B) C. briggsae JU1264 infected with SANTV JUv1264. (C) C. briggsae JU1264 infected with LEBV JUv1498. Note that the different graphs of small RNA distribution along the viral genome are not at the same scale. Because of the large number of reads mapping at the 5’ end of SANTV RNA1, the other reads along the molecule are here difficult to see. (TIF) [file ppat.1012259.s006.tif]
